# Highly Sensitive Shack–Hartmann Wavefront Sensor: Application to Non-Transparent Tissue Mimic Imaging with Adaptive Light-Sheet Fluorescence Microscopy

**DOI:** 10.3390/mps2030059

**Published:** 2019-07-11

**Authors:** Javier Morgado Brajones, Gregory Clouvel, Guillaume Dovillaire, Xavier Levecq, Corinne Lorenzo

**Affiliations:** 1 ITAV, Université de Toulouse, CNRS, 31106 Toulouse, France; 2 Imagine Optic, 91400 Orsay, France

**Keywords:** light-sheet fluorescence microscopy, adaptive optics, highly-sensitive Shack–Hartmann, tissue mimics

## Abstract

High-quality in-depth imaging of three-dimensional samples remains a major challenge in modern microscopy. Selective plane illumination microscopy (SPIM) is a widely used technique that enables imaging of living tissues with subcellular resolution. However, scattering, absorption, and optical aberrations limit the depth at which useful imaging can be done. Adaptive optics (AOs) is a method capable of measuring and correcting aberrations in different kinds of fluorescence microscopes, thereby improving the performance of the optical system. We have incorporated a wavefront sensor adaptive optics scheme to SPIM (_WAO_SPIM) to correct aberrations induced by optically-thick samples, such as multi-cellular tumor spheroids (MCTS). Two-photon fluorescence provides us with a tool to produce a weak non-linear guide star (NGS) in any region of the field of view. The faintness of NGS; however, led us to develop a high-sensitivity Shack–Hartmann wavefront sensor (SHWS). This paper describes this newly developed SHWS and shows the correction capabilities of _WAO_SPIM using NGS in thick, inhomogeneous samples like MCTS. We report improvements of up to 79% for spatial frequencies corresponding to cellular and subcellular size features.

## 1. Introduction

In recent years, tissue mimics (TMs) such as microtissues, spheroids, and organoid cultures have become increasingly important in life-science research, as they provide a physiologically more relevant environment for cell growth, tissue morphogenesis, and stem cell differentiation. Selective plane illumination microscopy (SPIM), the most prevalent light-sheet fluorescence microscope, is a sine qua non tool today to understand cell biology in these TMs. Yet, due to their thick, inhomogeneous, and high light-scattering properties, observing TMs at high resolution, in-depth, and in real time remains a major technical challenge. While SPIM is recognized as a very powerful and promising tool for imaging such samples [1], it still suffers from various specific limitations. Indeed, scattering, but also absorption and optical aberrations, limit the depth to which useful imaging can be done. Moreover, in these biological samples, refractive index differences in and between cells are the main source of optical aberrations [1,2,3]. Overall, these effects severely limit SPIM for imaging deep within complex heterogeneous opaque TMs, typically beyond 50–100 µm.

Previous approaches to improve in-depth image quality include the use of adaptive optics (AOs). Originally developed for astronomical telescopes, AOs is a method capable of measuring and correcting optical aberrations, thereby improving performance of the optical system. In direct wavefront sensing configurations, it does so by introducing a sensor, such as a Shack–Hartmann wavefront sensor (SHWS), that measures the distorted wavefront coming from an ideal point source emitter. In astronomy, this point source emitter can be a well-known star (a “guide star”) or the fluorescence produced when focusing a powerful pulsed laser into the atmosphere (artificial guide star). The guide star must be smaller than the diffraction limit defined by an SHWS microlens, and its emitted fluorescence must generate enough photons to provide the required signal-to-noise ratio for accurate wavefront measurements. From the wavefront measurement, a corrective element, such as a deformable mirror (DM), compensates for the optical aberrations by means of a feedback loop (e.g., a closed-loop).

Over the past decade, AOs has been increasingly used in microscopy and has proven a valuable tool for correcting aberrations in different kinds of fluorescence microscopes, such as confocal [4], two-photon fluorescence [5], structured illumination [6], and lattice light-sheet microscopes [7]. Wavefront sensor-less AOs has also been implemented in several microscope modalities [8,9], including light-sheet microscopy [10]. Previously, we successfully applied a SHWS-AOs scheme using fluorescent beads as guide stars [3] to use with SPIM (_WAO_SPIM) to correct complex aberrations induced by the TM itself. The same setup was also used to compensate for aberrations arising from refractive index mismatches induced by optical clearing methods [10]. Direct wavefront measurements using an SHWS in biological samples remain difficult since the latter do not display natural point source references, as do the guide stars used in astronomy.

In Jorand and coworkers [3], we addressed this issue by using bright 2.5 µm fluorescent beads directly inserted into the sample as suitable fluorescence point source references. This method enabled us to measure the aberrations induced by multi-cellular tumor spheroids (MCTS) and to compensate for them using closed-loop AOs. MCTS are TM models that are highly useful in investigating the influence of malignant cell interactions during cell proliferation [11]. Due to their opacity and density; however, they raise significant challenges for imaging by light microscopy [12]. Even if, in our case, the inserted fluorescence beads did not seem to alter the MCTS growth, they can disturb cell behavior within MCTS and; thus, the natural MCTS architecture. These possible effects need to be considered for live 3D imaging experiments. Moreover, spatial variations of aberrations within such inhomogeneous, non-transparent samples result in major changes to the light path and limit the quality of AOs correction outside a small region around the guide star—the isoplanetic patch. Guide star placement; therefore, plays a critical role in the AOs process. Bead placement inside living samples is random and intrusive [13], and requires the use of a pinhole limiting the usefulness of the correction.

To overcome these difficulties and to provide a minimally-invasive and more versatile approach, Aviles-Espinosa [8] and Wang [14] proposed the use of a non-linear guide star (NGS) that was successfully applied to confocal [15] and lattice light-sheet microscopes [7]. However, in samples such as MCTS, the NGS fluorescence light reaching the sensor is weak and often limits the accuracy of the wavefront measurement and; therefore, the efficiency of the correction. Indeed, light scattering drastically reduces the number of ballistic photons forming the NGS image, making it impossible to accurately measure aberrations with an SHWS coupled with a CCD sensor (as in our previous work). Speed of image acquisition with correction is limited by exposure time and by sampling requirements to accurately measure the wavefront from the guide star. Therefore, in live 3D imaging experiments where phototoxicity and speed are critical, the use of an NGS in combination with an SHWS coupled with a CCD sensor rapidly reaches its limits. Electron multiplying CCD (EMCCD) sensors offer high sensitivity and very low read-out noise, making them an ideal choice in this context. Recently, EMCCD-based SHWFS have been successfully employed in correcting sample-induced aberrations [7]. However, little detail has been published about the practical considerations of developing and using an EMCCD-based SHWFS. We have developed a high-sensitivity EMCCD-based SHWFS suitable for NGS and compatible with live 3D TM imaging studies. To be able to work with minimal light, the SHWFS development focused on photon collection efficiency, optimizing the microlens array to maximize photon flux in each sub-pupil of the sensor. A complete description of our sensor is presented in this article.

In optically-thick TMs, the loss of signal-to-background ratio due to scattering defines the maximum depth at which useful corrections can be made. Previous applications of AOs in lattice light-sheet microscopy show image quality improvements up to 30 µm deep [10] when imaging organoids. However, conventional light-sheet microscopy systems, including our own, typically entail imaging larger volumes, comprising larger fields of view, and requiring correction at greater depths. By coupling our _WAO_SPIM with an EMCCD-based SHWFS specifically designed for TM imaging, we demonstrate the in-depth correction capabilities in MCTS up to 120 µm deep.

## 2. Materials and Methods

### 2.1. Experimental Setup

Laser lines were generated using a compact laser launch (Errol). Laser beams of 491, 532, and 595 nm were generated and merged into a single beam. An acousto-optic tunable filter allowed for precise control over the illumination intensity of each beam.

The output beam was collimated and expanded by means of a telescope. A cylindrical lens generated the light-sheet which was focused into the sample with a 10 × 0.25 NA air objective (Leica Microsystems). The light-sheet thickness was 2.02 µm at the waist, with a Rayleigh range of 21.54 µm. Fluorescence light was collected by a 20 × 0.5 NA water immersion objective (Leica Microsystems) and expanded by telescope. The back-pupil plane of the telescope was conjugated with a Mirao 52-e DM (Imagine Optic, Orsay, France). The wavefront was measured using an SHWS made of a 14 × 14 microlens array and a Photometrics Evolve 512 EMCCD camera. In order to correct imaging path aberrations, the SHWS was first placed on the detection path. In this position, the wavefront was corrected to a plane wavefront, and the shape of the mirror was recorded. After correction, the SHWS was placed perpendicularly to the detection path. A dichroic beamsplitter (model FF520-Di02Semrock, Rochester, NY, USA) directed the 350–512 nm wavelengths to the SHWS while allowing the 528–950 nm wavelengths to reach the ORCA-D2 camera (Hamamatsu Photonics, Hamamatsu, Japan). The camera itself allowed for dual imaging in 641 (CCD1) and 520 nm (CCD2) wavelengths.

**Figure 1:**
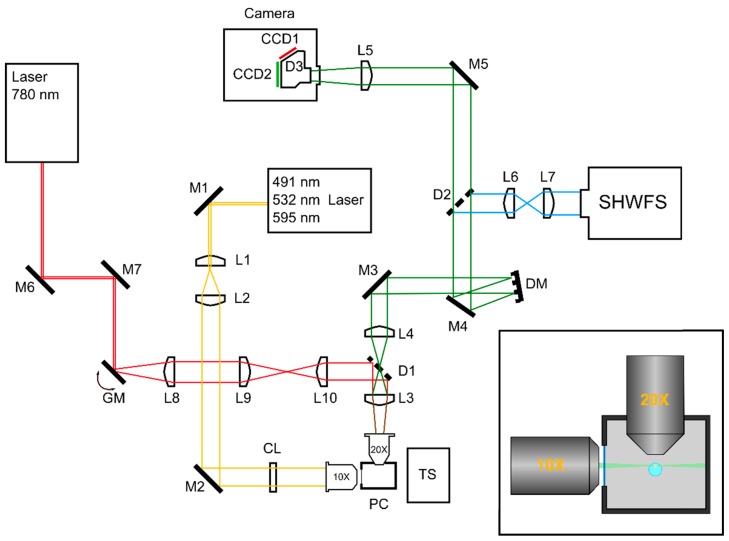
(**left**) Schematic image of the _WAO_SPIM system. L1–10: Lenses; M1–7: Mirrors; D1–3: Dichroic beamsplitters; CL: Cylindrical lens; DM: Deformable mirror; SHWFS: Shack–Hartmann wavefront sensor; GM: Pair of galvanic mirrors; PC: Physiological chamber; TS: Translation stage. (**right**) Top-down view of the sample mounting scheme. The sample is placed inside an agarose cylinder that hangs from a movable stage. The physiological chamber is filled with an aqueous solution.

A 780 nm femtosecond laser (Toptica Photonics, Gräfelfing, Germany) was directed to the sample by a pair of galvanometric mirrors. The galvanic mirrors were conjugated with the back-pupil plane of the detection objective. A dichroic mirror (model FF746-SDi01, Semrock, Rochester, NY, USA) made it possible to insert the infrared beam into the detection path, while the beam was focused into the sample by the detection objective. The sample was placed in the light-sheet inside a physiological chamber filled with aqueous solution. A moving stage enabled precise three-dimensional placement of the sample (Figure 1).

In _WAO_SPIM, dichroic beamsplitters D1 and D2 introduce significant aberrations, mostly astigmatism and coma. To compensate for these optically-induced aberrations, the latter were measured by placing the SHWS in the imaging camera focal plane. We could then set the DM shape (“AOs off” in the text) so that it corrected imaging path aberrations. By repositioning the SHWS in its dedicated path, we measured a reference wavefront corresponding to the differential aberrations between the imaging and the SHWS path. This reference wavefront was henceforth used as a target for all AOs corrections.

The optical system was driven by WaveSuite software (Imagine Optic) and custom-made AMISPIM software.

### 2.2. Cell Culture and MCTS Production

HCT116-H2BGFP/ArrestRed cells were cultured in DMEM+GlutaMAX (Dulbecco’s modified eagle medium; Thermo Fisher Scientific, Waltham, MA, USA), supplemented with 10% fetal bovine serum and 1% penicillin–streptomycin (Pen Strep; Gibson), and maintained at 37 °C with 5% CO_2_ in an incubator. To produce the MCTS, we used the centrifugation method described in [16]. MCTS were prepared in ultra-low attachment 96-well plates (Costar). Cells were plated at a density of 1000 cells/well in 150 µL cell culture medium per well, and then centrifuged to enable MCTS formation. After three to four days of growth, MCTS of 300 µm in diameter were collected, washed three times with PBS, before being fixed with 10% neutral buffered formalin (Sigma-Aldrich) at room temperature for two hours.

### 2.3. Sample Preparation

2.5 µm fluorescent beads (Invitrogen Inspeck Green 505/515 100% relative intensity) and MCTS were embedded in 1.3% agarose (Euromedex low melting point) inside 100 µL capillary pipettes (Hirschmann Ringcaps).

### 2.4. Image Processing

Images were processed with open-source image-processing Fiji software and custom MATLAB scripts. Image quality assessment (spatial frequency comparison) Matlab codes are available on request.

## 3. Results

### 3.1. Wavefront Sensor

Our SHWS is based on a Photometrics Evolve 512 EMCCD camera. With a quantum efficiency greater than 0.9 in the visible range, EMCCD technology provides the most sensitive sensors available. The sensor’s electromagnetic gain can be tuned in real time allowing a trade-off between exposure time and signal-to-noise ratio. These characteristics make EMCCD a versatile tool when working with a low photon budget, and a good candidate for a high sensitivity wavefront sensor. The Evolve 512 EMCCD camera has a 1–300 EM gain range.

**Figure 2:**
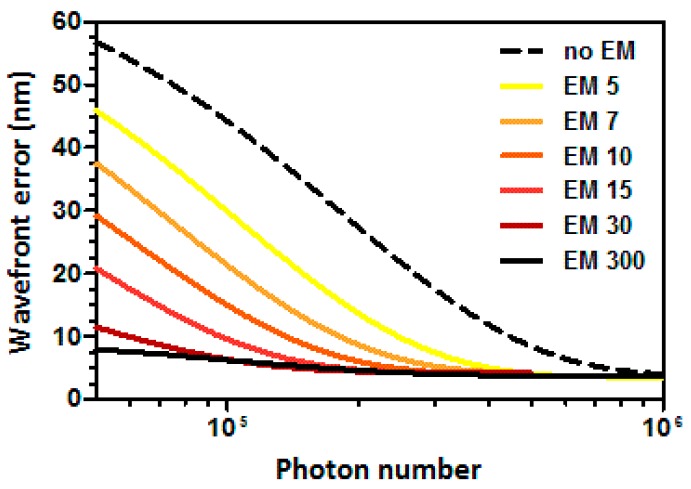
Simulated wavefront error as a function of the total number of photons arriving at the sensor.

Wavefront measurement error depends on photon flux and EM gain. Wavefront reconstruction in a SHWS is based on the correct centroid calculation of the Shack–Hartmann spots and thus amplification of noise, if too strong, may introduce errors. We modeled the wavefront error as a function of photon flux and EM gain. This analysis concludes that we need at least $2\times 10^{5}$ photons for an optimal wavefront measurement (Figure 2). With $2\times 10^{5}$ photons and a gain of 10 or more, the wavefront error is kept below 7 nm. Additionally, using a gain of 300 allows us to work with less than $5\times 10^{4}$ while maintaining error of less than 10 nm.

**Figure 3:**
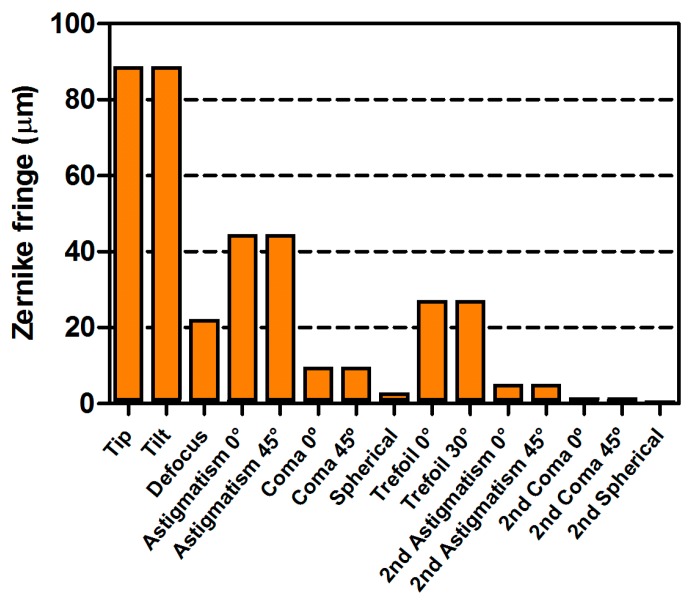
Simulated dynamic range of wavefront reconstruction.

Wavefront sensor design is a multiparameter problem leading to a compromise between different variables concerning the microlens matrix design (focal length, size, distribution) and camera specifications (pixel size, CCD surface). All of these parameters must be finely tuned to be able to measure local wavefront slopes according to the application requirements. Table 1 presents details of the sensor properties. The most significant aberrations in SPIM for MCTS imaging with minimized refractive index mismatch conditions, due to the sample itself, can be decomposed primarily as astigmatism, coma, and spherical aberrations themselves. To help define optimal wavefront sensor design, we performed a simulation (data not shown) of the wavefront measurement dynamic and accuracy as a function of the number of microlenses and photon flux. Wavefront correction error is dependent on both the wavefront measurement and wavefront modulation errors. To ensure accurate wavefront correction by the DM, wavefront measurement error must be kept smaller than the modulation error (for the Mirao 52-e mirror, no more than 20 nm rms, cf. Mirao 52-e specification sheet). This accuracy must be kept constant for different levels of aberrations to obtain reliable correction quality. A tradeoff must; therefore, be made between the necessary photon flux and the sensor-measured amplitude of the aberrations: When compared to conventional SHWS design typically used in metrology, the numerical aperture of the microlenses was slightly increased (~25%) in order to boost photon collection efficiency, while, as a consequence, spatial sampling was slightly decreased. Based on previous MCTS experiments, we evaluated that a 3 µm peak-to-valley dynamic range is necessary on the third-order spherical aberration ($Z_{4}^{0}$) for a 555 nm wavelength. Using these criteria, a 14 × 14 microlens array is able to provide optimized wavefront reconstruction. Figure 3 presents an evaluation of the sensor dynamic range.

### 3.2. System Performance

We first measured the performance of our system by evaluating its correction capabilities using fluorescent beads in a transparent environment. We directly measured RMS aberrations and calculated the Strehl ratio using the Maréchal approximation. To evaluate the amount of light reaching the sensor, the number of photons was estimated from the physical properties of the sensor as follows:

$$N_{photons}=\frac{I_{signal}\cdot CG}{EMG\cdot QE},$$

where $N_{photons}$ is the number of signal photons over the pupil, $I_{signal}$ is the sum of the pixel value over the image after removing background, CG is the conversion gain of the sensor, EMG is the electromagnetic gain applied, and QE is the quantum efficiency of the sensor.

**Figure 4:**
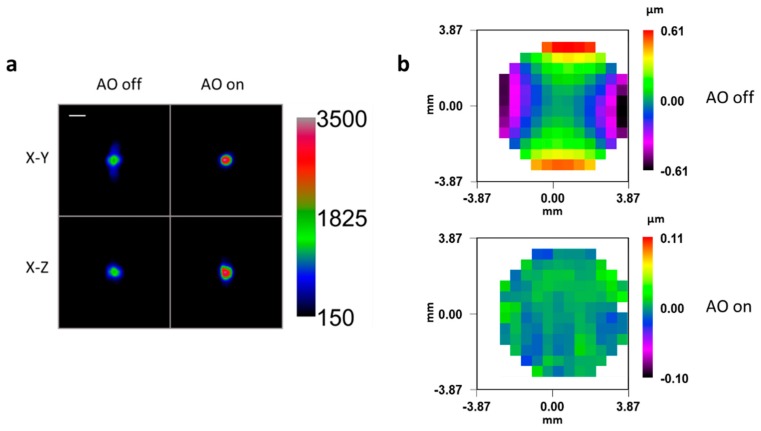
(**a**) Maximum intensity projection of green fluorescent beads embedded in 1% agarose and imaged through a capillary glass at a depth of 100 µm, both before and after Adaptive Optics (AO) correction. Scale bar: 5 µm. Beads were illuminated with constant intensity at 491 nm. (**b**) Measured wavefronts before and after correction, offset by the reference wavefront corresponding to an aberration-free system.

Green fluorescent beads were suspended in agarose inside a glass capillary. The latter acted as a source of aberration, whose main component was astigmatism and to a lesser extent trefoil and first order coma. The bead image appears significantly distorted in lateral and axial directions (Figure 4).

The image was correctly restored after AOs. RMS error was reduced from 0.238 ± 0.014 µm to 0.011 ± 0.001 µm, and the Strehl ratio improved from 0.007 ± 0.021 to 0.983 ± 0.005 after correction. Contrast was improved by an average 80% ± 29% (peak signal to background). Full width at half maximum (FWHM) values before and after correction are shown in Table 2. Note that FWHM_Z_ depends on the thickness and optimal placement of the light-sheet and thus does not improve.

**Figure 5:**
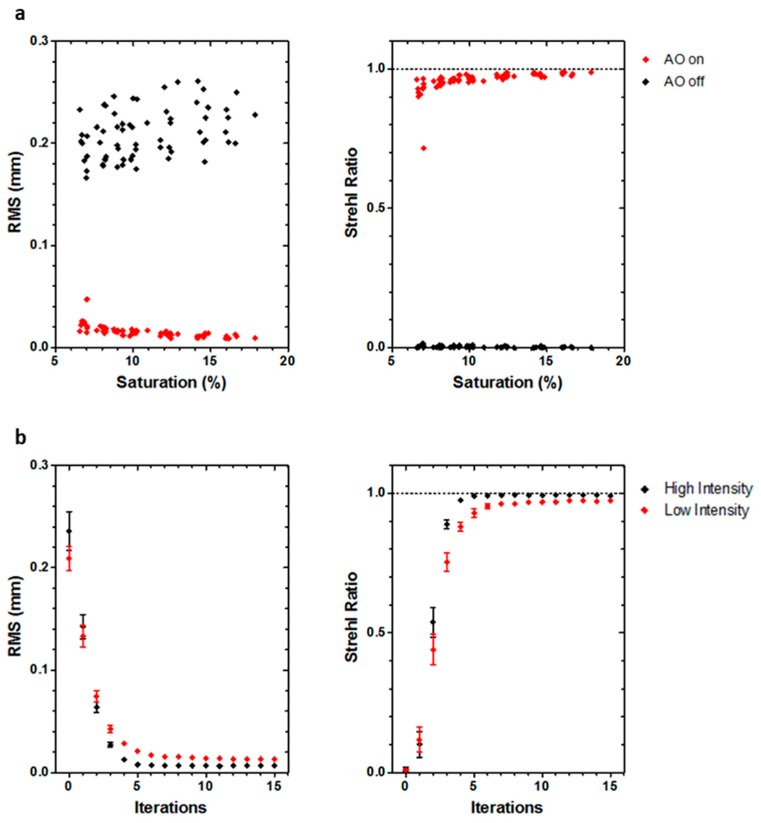
(**a**) RMS and Strehl ratio values of wavefronts before (AOs off) and after (AOs on) correction for a range of light conditions. Each pair of points (off and on) corresponds to a different fluorescent bead. (**b**) SR and RMS values after correction as a function of the number of iterations used in the closed loop, for both low (8% saturation) and high (90% saturation) emission intensity.

In order to determine the lowest limit in light intensity necessary to perform correction, fluorescent beads were placed inside glass capillaries and imaged at 100 µm in a similar manner as described above, with decreasing illumination intensity. During experiments, emission intensity was measured as a fraction of the saturation value of the SHWS, defined as the maximum readable intensity for each pixel. The number of captured photons was later calculated from the acquired SHWS images. Corrected images typically nearly reached the diffraction limit (SR>0.8) (Figure 5). Good corrections were obtained with as few as $12\times 10^{3}$ signal photons over the pupil, with correction performance increasing with intensity. For intensities below $12\times 10^{3}$ photons (6.6% of SHWS saturation value), our software failed to measure wavefront aberrations, making it impossible to correct the image. Beyond that limit, corrections typically allowed for imaging near the diffraction limit. Four closed loop iterations were necessary for good correction in low intensity conditions (8% of SHWS saturation value, $14.5\times 10^{3}$ to $16.5\times 10^{3}$ signal photons), while three were enough under high intensity conditions (90% of SHWS saturation value, $290\times 10^{3}$ to $305\times 10^{3}$ signal photons) (Table 3, Figure 5).

### 3.3. MCTS Imaging

To evaluate the ability of _WAO_SPIM to correct aberration within MCTS, we used MCTS prepared from the HCT116 cell line expressing H2B-GFP and ArrestRed (H2B-tKR, Evrogen, Moscow, Russia) histones, and fixed at approximately 300 µm size. The ArrestRed fluorophore was used for imaging purposes, while GFP was used for NGS generation. Image quality improvements were quantitatively assessed in terms of spatial frequency using a frequency metric presented in Masson and coworkers [10]. Frequencies were evaluated by integrating concentric rings into the Fourier space, thereby obtaining a radial frequency distribution. This process was carried out for before and after correction images in order to assess improvements in frequencies corresponding to features of cellular and subcellular size.

Although fluorescent beads are effective tools in characterizing the correction capability of our microscope, their usefulness is severely limited in living samples due to random spatial placement and intrusiveness. To overcome this limitation, an NGS was generated by excitation of the H2B-GFP nuclear protein by a 780 nm femtosecond laser.

**Figure 6:**
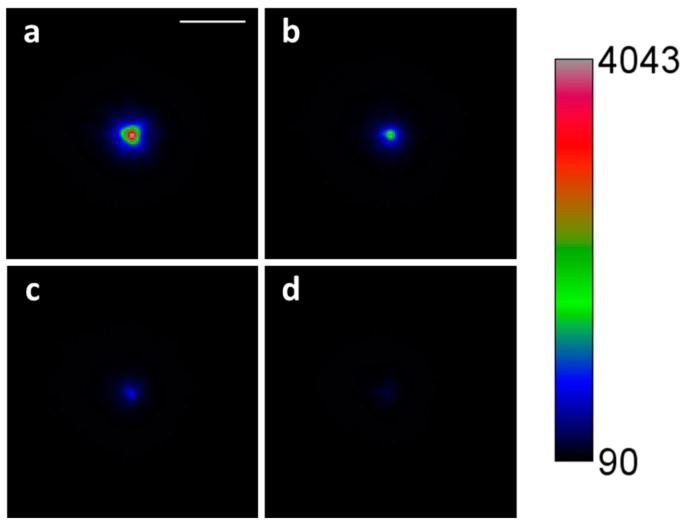
Widefield image of the guide star at different depths inside a multi-cellular tumor spheroid (MCTS). The MCTS was immersed in a fluorescein solution and the guide star imaged after 1 h. (**a**) Guide star outside spheroid. (**b**) Depth: 25 µm. (**c**) Depth: 50 µm. (**d**) Depth: 75 µm. Scale bar: 10 µm.

Firstly, we characterized the properties of the NGS. In order to decouple NGS shape from the fluorophore spatial distribution, we used non-labeled MCTS immersed in a fluorescein solution. The latter is a fluorophore that can be used for two-photon excitation with peak emission around 520 nm, and is; thus, suitable for NGS generation. Fluorescein quickly permeates MCTS, making it possible to generate an NGS within. The NGS was observed at different depths (Figure 6). Table 4 presents the observed size and peak intensity of the NGS. The incapacity of our system to move the guide star in the z direction prevented us from assessing the axial FWHM. The observed quality of the guide star decreases with depth, with fast deterioration occurring typically around the 70 µm mark. Note that this decrease in observed quality is due to aberration and scattering in both the illumination and detection paths.

**Figure 7:**
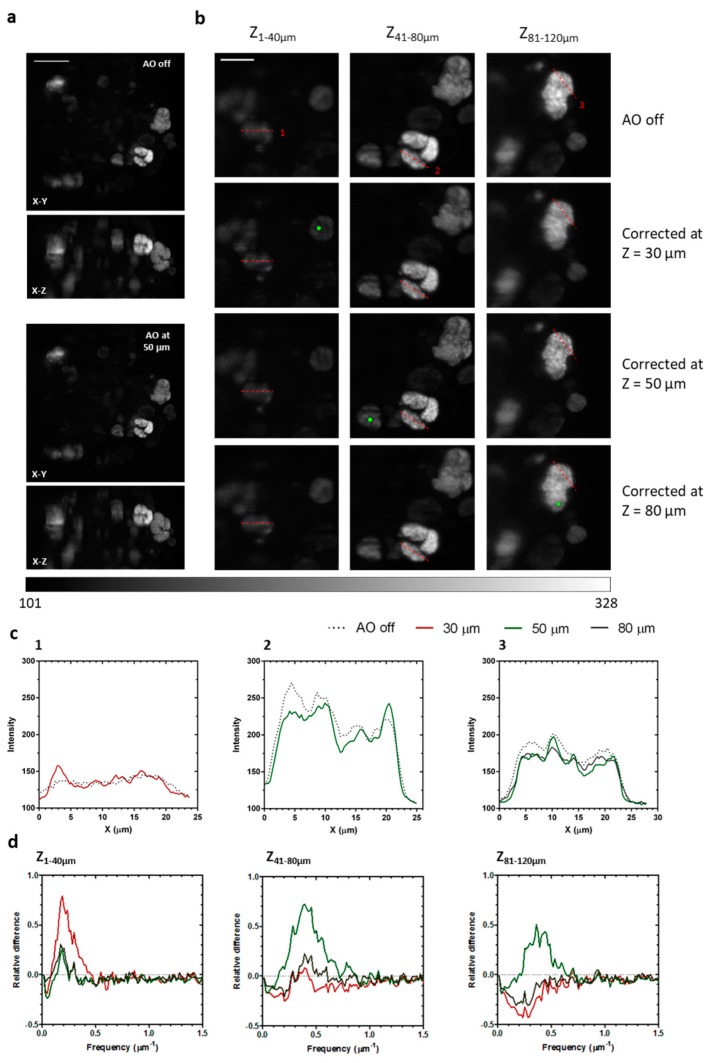
Image quality improvement in depth. (**a**) Maximum intensity projection of MCTS expressing the fluorescent protein H2B-ArrestRed, before AOs correction and after correction at 50 µm. Scale bar: 50 µm. (**b**) Maximum intensity projections of three regions of interest before AOs correction and after correction at 30, 50, and 80 µm. NGS were placed at the green dot in the respective corrections. All images were acquired using the same excitation intensity and 100 ms exposure. Corrections were performed using 1 s exposures. Scale bar: 25 µm. (**c**) Intensity profiles along paths 1, 2, and 3. (**d**) Relative differences of spatial frequencies between each of the corrections and the uncorrected case for each region of interest.

MCTS were mounted inside agarose cylinders and observed before and after AOs correction (Figure 7). Corrections were performed at different depths, and MCTS were imaged each time up to a depth of 120 µm. In order to evaluate image quality at different depths, the 3D image was divided into three Z sections (Z_1–40µm_, Z_41–80µm_, Z_81–120µm_), after which a maximum intensity projection was performed in each one. Typically, a maximum of five to ten closed loop iterations could be performed due to the fast bleaching of the fluorophore and the long exposure times required for wavefront measurement (usually 0.3 to 1 s).

The AOs-corrected images show improvements in contrast and resolution when compared to uncorrected ones. Subcellular sized structures that were not well resolved in the “AOs off” position could be resolved after correction (Figure 7a–c).

Spatial frequencies were evaluated by means of integrating the radial frequencies along concentric rings centered at the zero frequency (Figure 7d). Compared to the uncorrected case, there is a substantial increase (up to 79%) of frequencies in the range 0.1 to 0.8 µm^−1^, corresponding to features of cellular and subcellular size (1.25 to 10 µm). Frequencies outside this range showed a slight decrease. At the Z_1–40µm_ section, the most efficient correction was realized at 30 µm, while at the Z_41–80µm_ section the most efficient was realized at 50 µm. At Z_81–120µm_ imaging depth, correction becomes unreliable and those performed at 30 and 80 µm showed a general worsening of image quality, although the one performed at 50 µm showed a slight improvement. Peak improvement in image quality, then, is greater for corrections realized at lower depths.

## 4. Discussion and Conclusions

Biological tissue can be difficult to image in depth due to absorption, scattering, and aberrations originating from both the sample and the optical system. SPIM is a popular in-depth imaging tool that has been coupled with adaptive optics to improve imaging by correcting system and sample-induced aberrations, most common in transparent tissue. However, scattering poses a challenge for AOs correction, compounded by the difficulty of adequate guide star generation within optically-thick samples.

We opted for a non-linear guide star approach, using fluorescent labeled proteins to create a point-like source of light through two-photon excitation. This approach is compatible with live samples and requires little sample preparation. However, the low intensity of the non-linear guide star requires the use of custom-built sensors. We; thus, developed and described an EMCCD-based SHWS to correct transparent inert samples using only 12,000 photons. Nonetheless, in order to measure the wavefront from inside non-transparent live samples such as MCTS, second-long exposure times are often required. When coupled with fast bleaching; however, this severely limits the number of correction steps possible (although four usually suffice). It is our belief that limiting the correction to the main modes of aberration induced by the sample could lessen the number of iterations required and increase correction reliability, while still providing significant image quality improvement.

We have shown improvements in image quality of optically-thick fixed samples, as per resolution and high-frequency detail. Corrections are especially effective in shallow depths (less than 50 µm), although image quality can be improved as deep as 120 µm. This represents an improvement over previously reported AOs implementations in light-sheet systems when imaging scattering samples. Indeed, correction depth is mainly limited by scattering: Signal-to-noise ratio becomes increasingly worse with depth due to the loss of ballistic light and the increment of noise due to scattered light, hindering centroid calculation of the Shack–Hartmann spots, and causing loss of wavefront measurement accuracy. Therefore, the use of longer wavelength NGS, less sensitive to scattering, could increase useful correction depth. Since aberrations are spatially dependent, a single correction usually does not improve the whole image. A scanning algorithm has been proposed in order to perform an averaged correction and reduce scattering artifacts when measuring the wavefront [7]. While it could be implemented in our system (we expect it to have a limited effect in our case), it does; however, slightly increase system complexity. Another possible improvement could be implementing a tiling algorithm in order to correct different regions of interest that could then be processed to obtain a single reliably-corrected image. This tiling approach; however, would require increasing correction time by at least one order of magnitude, thereby limiting temporal resolution. In SPIM, scattered illumination light gives way to a decrease in the penetration depth of the light-sheet, and results in ghost image artifacts. Scanned Bessel beams have shown increased penetration depth [17] and reduction of ghost artifacts thanks to the self-healing nature of the beam [18]. The use of an alternative illumination strategy, such as a scanned Bessel beam, could further improve detection of features deep into the sample [6].

In conclusion, _WAO_SPIM is a powerful tool for in-depth imaging in thick non-transparent TMs, and we are confident that future development will resolve remaining shortcomings.

## Figures and Tables

**Table 1:** Technical characteristics of our Shack-Hartmann Wavefront Sensor.

**Aperture Dimension (mm^2^)**	**7.73 × 7.73**
Number of microlenses (µm)	14 × 14
Microlens step (µm)	0.552
Focus dynamic range (mm)	24.1
Wavefront dynamic on $Z_{0}^{4}$	5.4 λ
Photon number for optimal accuracy	200,000
Wavefront measurement accuracy	λ/70
Working wavelength range (nm)	400–800
Readout speed (MHz)	5
Electron-Multiplying gain	1–300

**Table 2:** Mean Full width at half maximum (FWHM), root mean square aberration (RMS), and Strehl ratio (SR) values for beads before and after correction.

	AOs off	AOs on
FWHM_X_ (µm)	3.376	2.607
FWHM_Y_ (µm)	4.679	2.426
FWHM_Z_ (µm)	3.486	3.781
RMS (µm)	0.238	0.011
SR	0.007	0.983

**Table 3:** Mean correction results for different emission intensities.

	AOs on			
	7% ^1^	8%	9%	10%
FWHM_X_ (µm)	2.672 ± 0.019	2.695 ± 0.016	2.686 ± 0.018	2.682 ± 0.018
FWHM_Y_ (µm)	2.343 ± 0.030	2.230 ± 0.045	2.277 ± 0.030	2.281 ± 0.025
FWHM_Z_ (µm)	3.491 ± 0.073	3.483 ± 0.065	3.495 ± 0.070	3.540 ± 0.063
RMS (µm)	0.025 ± 0.002	0.016 ± 0.001	0.014 ± 0.001	0.013 ± 0.001
SR	0.909 ± 0.011	0.961 ± 0.003	0.969 ± 0.003	0.973 ± 0.002

^1^ Intensity was measured as a fraction of the saturation intensity of the sensor. Wavefront information was acquired at 100 ms exposure time and EM gain of 300. Fifteen iterations were used to perform the corrections. Illumination intensity was adjusted on a case-by-case basis to achieve the desired emission intensity.

**Table 4:** FWHM of the non-linear guide star at different depths inside the MCTS.

Depth (µm)	FWHM_X_ (µm)	FWHM_Y_ (µm)
0	2.23	2.18
25	2.44	2.14
50	2.38	2.27
75	4.74	4.89
100	8.65	7.90
